# *Crocus sativus* L. Extract and Its Constituents: Chemistry, Pharmacology and Therapeutic Potential

**DOI:** 10.3390/molecules26144226

**Published:** 2021-07-12

**Authors:** Nikolaos Pitsikas, Konstantinos Dimas

**Affiliations:** Department of Pharmacology, Faculty of Medicine, School of Health Sciences, University of Thessaly, Biopolis, Panepistimiou, 341500 Larissa, Greece

Natural products or organic compounds isolated from natural sources as primary or secondary metabolites have inspired numerous drugs. It is not an overstatement that the majority of medicines in clinics, even in the 21st century, have been derived from natural resources despite the declining of industry research into natural products due to a variety of drawbacks [1].

Saffron crocus, considered to be the most valuable spice by weight [2], and its bioactive constituents, have been reported to have been studied for the treatment of a wide range of pathologies including neuropsychiatric and neurodegenerative disorders, cancer, diabetes, and even cardiovascular diseases.

In this special issue on the chemistry, pharmacology, and therapeutic potential of *Crocus sativus* L. extract and its constituents, two reviews report new data and advances in the fields of schizophrenia and cancer. In the first review, Pitsikas critically assesses advances in the research of these molecules for therapy for schizophrenia, a chronic, mentally devastating disease [3]. In the second review, Labrianidou and her colleagues provide an insight into the advances in research on the anticancer properties of saffron and its components, discussing preclinical data, clinical trials, and patents aiming to improve the pharmacological properties of saffron and its major ingredients [4].

This special issue of *Molecules* aims furthermore to assess new advances in the understanding of the therapeutic action of saffron and its constituents in targeting different pathologies. In this context, eight original research articles covering some recent advances in the therapeutic actions of saffron and its ingredients in different diseases are reported herein. Amin et al. present new data on the effects of saffron and its major ingredients, safranal and crocin, on colon cancer cells with mismatch repair (MMR) deficiency and microsatellite instability. In this study, saffron and its components are reported to show a significant anti-proliferative effect in cells with deficient MMR [5]. In another interesting work, Suhrid Banskota et al. present data which suggest that pre-treatment with saffron inhibits dextran sulfate sodium (DSS)-induced pro-inflammatory cytokine secretion; modulates gut microbiota composition; prevents the depletion of short-chain fatty acids (SCFA) such as isobutyric acid, acetic acid, and propionic acid; and reduces susceptibility to colitis, a result of special interest, as long-standing colitis is well-known to be associated with increased risk of colon cancer [6]. Four articles report novel findings on the effects of saffron and its ingredients on neuropsychiatric disorders. In the first of two articles, Pitsikas and Tarantilis report the beneficial effects of crocins on memory loss, induced by the widely used anesthetic ketamine, in rats [7]. In the second article, the same authors find that the anxiolytic properties of crocins are mediated by their agonistic action on the GABA_A_-benzodiazepine receptor [8]. Orio et al. evidence the antidepressant effects of a standardized saffron extract, Affron®, suggesting that oral saffron may exert a beneficial action in anxious and depressive states [9]. The collection of the articles reporting new findings on neurological disorders is filled in nicely with a work by Maggi and colleagues, demonstrating the ability of saffron to cope with retinal neurodegeneration, and this beneficial action appears to be dependent on the presence of specific crocins and the contribution of other saffron components [10].

The interesting results of two studies performed by Rossi and colleagues [11] and Kakouri et al. [12] corroborate further for the therapeutic potential of saffron. Specifically, Rossi et al. demonstrate the efficacy of crocetin in alleviating irradiation injury in an in vitro model of the pubertal testis, suggesting the therapeutic potential of this bioactive constituent of saffron as a fertoprotective agent against ionizing radiation’s deleterious effects in the pubertal period [11].

Additionally, Kakouri and her colleagues, in a nice, in vivo study conducted on the zebrafish, demonstrate that that the application of crocins reduces glucose levels in the zebrafish embryo and enhances insulin expression. They further show that following a single administration of crocins, the expression of phosphoenolpyruvate carboxykinase1 (pck1), a key gene involved in glucose metabolism, is increased, which is indicative of a putative role for crocins in glucose metabolism and insulin management [12].

The collection of this special issue is completed with two articles on two novel methods regarding the bioanalysis of saffron extracts. Gikas et al. report the use of high-resolution mass spectrometry metabolomics studies as novel tools to develop a fingerprint of the various saffron extracts which obviously, given the health-promoting effects of the extracts, can be of great importance for the selection of the appropriate saffron sample [13]. Finally, Girme et al. report the development and application of the pharmacokinetics of a new bioanalytical method based on a sensitive and ultra-fast liquid chromatography (UFLC)-tandem mass spectrometry method with high assay-based precision and accuracy on analytical quality control levels and excellent recoveries in plasma samples [14]. These latter results, as the authors state, suggest that this new procedure may be an appropriate bioanalytical method for preclinical/clinical trials on *Crocus sativus* extract’s main ingredients.

In conclusion, in this special issue of *Molecules*, we are delighted to have received several works that we hope provide new and interesting information for the scientific community on the chemistry, pharmacology, and therapeutic potential of *Crocus sativus* L. extract and its constituents. 
